# Immunotherapy in Acute Myeloid Leukemia: Where We Stand

**DOI:** 10.3389/fonc.2021.656218

**Published:** 2021-05-10

**Authors:** Alessandro Isidori, Claudio Cerchione, Naval Daver, Courtney DiNardo, Guillermo Garcia-Manero, Marina Konopleva, Elias Jabbour, Farhad Ravandi, Tapan Kadia, Adolfo de la Fuente Burguera, Alessandra Romano, Federica Loscocco, Giuseppe Visani, Giovanni Martinelli, Hagop Kantarjian, Antonio Curti

**Affiliations:** ^1^ Haematology and Stem Cell Transplant Center, AORMN, Pesaro, Italy; ^2^ Hematology Unit, Istituto Scientifico Romagnolo per lo Studio e la Cura dei Tumori (IRST) IRCCS, Meldola, Italy; ^3^ Department of Leukemia, MD Anderson Cancer Center, Houston, TX, United States; ^4^ MD Anderson Cancer Center, Madrid, Spain; ^5^ Dipartimento di Chirurgia e Specialità Medico-Chirurgiche, Sezione di Ematologia, Università degli Studi di Catania, Catania, Italy; ^6^ IRCCS Azienda Ospedaliero-Universitaria di Bologna, Istituto di Ematologia “Seràgnoli”, Bologna, Italy

**Keywords:** acute myeloid leukemia, tolerance, natural killer, immunotherapy, monoclonal antibody, checkpoint inhibitors

## Abstract

In the past few years, our improved knowledge of acute myeloid leukemia (AML) pathogenesis has led to the accelerated discovery of new drugs and the development of innovative therapeutic approaches. The role of the immune system in AML development, growth and recurrence has gained increasing interest. A better understanding of immunological escape and systemic tolerance induced by AML blasts has been achieved. The extraordinary successes of immune therapies that harness the power of T cells in solid tumors and certain hematological malignancies have provided new *stimuli* in this area of research. Accordingly, major efforts have been made to develop immune therapies for the treatment of AML patients. The persistence of leukemia stem cells, representing the most relevant cause of relapse, even after allogeneic stem cell transplant (allo-SCT), remains a major hurdle in the path to cure for AML patients. Several clinical trials with immune-based therapies are currently ongoing in the frontline, relapsed/refractory, post-allo-SCT and minimal residual disease/maintenance setting, with the aim to improve survival of AML patients. This review summarizes the available data with immune-based therapeutic modalities such as monoclonal antibodies (naked and conjugated), T cell engagers, adoptive T-cell therapy, adoptive-NK therapy, checkpoint blockade *via* PD-1/PD-L1, CTLA4, TIM3 and macrophage checkpoint blockade *via* the CD47/SIRPa axis, and leukemia vaccines. Combining clinical results with biological immunological findings, possibly coupled with the discovery of biomarkers predictive for response, will hopefully allow us to determine the best approaches to immunotherapy in AML.

## Introduction

Acute myeloid leukemia (AML) is a clonal disease characterized by the rapid proliferation of immature myeloid cells in the bone marrow with impaired differentiation (1). Despite major progresses in AML therapy and high rates of complete remission (CR) after intensive chemotherapy, many patients will eventually relapse and die from the disease. At present, there is no curative therapy for patients who relapse, except for a small proportion who are cured by allogeneic stem cell transplant (allo-SCT) in second CR. The prognosis is even worse in elderly patients, where overall survival (OS) at 1 year is approximately 10–15%, due to the higher prevalence of unfavorable biological factors, such as poor risk cytogenetics and presence of unfavorable molecular aberrations coupled with poor tolerance to intensive therapies and higher frequency of concurrent comorbidities (1).

Recently, the identification of disease-specific molecular aberrations has triggered the development of targeted therapies (e.g.: FLT3 inhibitors, IDH1/2 inhibitors), to improve the clinical outcome of AML patients (2, 3). Furthermore, the new frontier of BCL-2 inhibition with venetoclax has brought new hopes, with improved CR rates, prolonged event-free survival (EFS) and OS in the newly diagnosed elderly population (2, 4–7). Nevertheless, relapse still remain a major flaw, on the one hand due to the clonal evolution of the disease leading to disease recurrence (1), on the other hand due to the immunosuppressive milieu of the bone marrow microenvironment leading to immune escape (4, 8–11).

In recent years, the importance of the fine-tuned interplay between AML blasts, the hematopoietic *niche*, and the cells of the immune system for AML development and growth has been demonstrated (8, 10). We have better understood the mechanisms underlying the ability of AML cells to induce immunological escape and systemic tolerance (9, 10). Such tolerogenic pathways, which create an immunosuppressive microenvironment, have been suggested to both critically hamper anti-leukemia immune responses and to negatively impact the anti-leukemia efficacy of conventional and experimental therapies. Clinically relevant, some of these pathways could be targeted by new drugs, such as immune checkpoint and macrophage checkpoint inhibitors, resulting in increased anti-tumor immunity.

Given the molecular heterogeneity of AML (9) and the natural diversity of AML blasts, the best strategy to beat AML will eventually be to combine or sequentially apply chemotherapy, immunotherapy and molecular therapy, designing a three-headed chimera hopefully able to control the disease and avoid its recurrence. Moreover, the use of novel techniques for monitoring of the minimal residual disease (MRD), to identify possible recurring clones at an early time-point, together with biomarkers of early response and genomic profiling (12), are required to increase the cure rate of AML.

This review summarizes the available clinical data with immune-based therapeutic modalities such as monoclonal antibodies, T cell engager antibodies, alloreactive NK and CART cell, immune checkpoint blockade *via* blockade of PD-1/PD-L1, CTLA4, TIM3 and macrophage checkpoint blockade *via* the CD47/SIRPa axis.

## Monoclonal Antibodies

CD33, CD123, CLL1, TIM3, and CD244 are ubiquitously expressed on AML bulk cells and, often, in leukemic stem cells (LSCs), both at diagnosis and relapse, irrespective of genetic characteristics and leukemic clonal evolution. Accordingly, they have been considered as ideal targets for AML immunotherapy (13).

Monoclonal antibodies treatments include naked antibodies against AML surface antigens such as CD33 (e.g. lintuzumab) or CD38 (e.g. daratumumab), antibodies conjugated to toxins in various anti-CD33 (gemtuzumab ozogamicin, SGN33A, IMGN779) and anti-CD123 (IMGN632, SL-401, SGN-CD123A) formulations, antibodies conjugated to radioactive particles such as Iodine or Actinium-labeled anti-CD33 or anti-CD45 antibodies, and multiple bispecific antibodies (**Table 1**).

**Table 1:** Monoclonal antibodies in AML.

Category	Drug name	Drug details	Antigen/target	Combination therapy	Indication (AML only)	Effects *in vitro o vivo*	Refs
**Unconjugated Monoclonal antibody**	Lintuzumab (HuM195)	Monoclonal Antibody	CD33	Salvage chemotherapy; cytarabine	R/R AML	Induced antobody-dependent cell-medated citotoxicity and fixing human complement *in vitro*; no improved in ORR or OS	(26–29)
	Talacotuzumab (CSL362)	Monoclonal Antibody	CD123	Daunorubicin and cytarabine; alone; decitabine	R/R AML; high risk AML in I or II CR; alderly high risk AML after HMA failure	Improved ORR; in elderly high-risk AML no efficacy	(46–51)
	CSL360	Chimeric Monoclonal Antibody	CD123	Alone	Advanced AML	No efficacy	(45)
**Conjugate antibody**	Gentuzumab ozogamicin (GO)	ADC combining calicheamicin-γ1 with a IgG4	CD33	Induction/consolidation	AML *de novo* with favorable and intermediate-risk	Improved EFS and OS	(14–19)
	Lintuzumab 213Bi	Abs labeled with the α-emitters bismuth- 213 (213Bi)	CD33	After chemotherapy	*De novo* or R/R AML	High toxicity and treatment-related death	(28)
	Lintuzumab Ac225	Abs labeled with the α-emitters actinium-225 (225Ac)	CD33	Salvage chemotherapy; venetoclax	R/R AML as a bridge to SCT	Improved CR/CRi rate with prolonged myelosuppression	(30, 31)
	SGN-CD33A	antibody-drug conjugate (ADC)	CD33	Alone; azacitidine	Relapsed CD33 positive or declined intensive therapy	Reduction in BM blasts; in combination early mortality	(33)
	Tagraxofusp (SL-401)	CD123-directed cytotoxin	CD123	Consolidation Therapy; azacitidine	Adverse risk AML in first CR or RR and naive AML not eligible for induction chemotherapy	Efficacy data not available	(41–44)
	IMGN632	ADC	CD123	Alone; azacitidine and/or venetoclax	R/R AML or R/R BPDCN	Improved CR/CRi in frontline BPDCN	(52–54)
**Bispecific antibodies**	AMG330	BiTE and CiTE	CD3 and CD33	Pembrolizumab;	R/R AML	*In vivo* prolonged OS; improved CR/CRi	(64–69)
	AMG673	BiTE	CD3 and CD33x	Alone	R/R AML	Improved CR/CRi	(70–72)
	G333	TandAbs	CD3 and CD33	Alone	R/R AML	Data not available	(73, 74)
	AMV564	TandAbs	CD33 and CD3	Pembrolizumab	R/R AML	Improved CR/CRi	Na
	Flotetuzumab (MGD006 or S80880)	DARTs	CD3 and CD123		R/R AML	Improved CR/CRi in primary refractory also in TP53 mutated	(75, 78–82)
	Vibecotamab (XmAb14045)	DARTs	CD3 and CD123	Alone	R/R AML heavily pretreated	Good efficacy	(84)
	SPM-2	TRiKe	CD33, CD123 and CD16	Alone	*In vitro* in blasts of unfavorable genetic subtype AML	Eliminate LSC	(85)

### Targeting CD33, the First Monoclonal Antibody-Based Strategy in AML

An increasing number of different types and classes of monoclonal antibodies have proven their efficacy and have been approved for indications in several hematological malignancies, comprising AML. In 2000, gemtuzumab ozogamicin (GO), a humanized anti-CD33 monoclonal antibody conjugated with calicheamicin, was granted accelerated approval by the United States (US) Food and Drug Administration (FDA) (14). However, the promising results of the phase II study conducted in relapsed, older adults with AML (15, 16) were not confirmed in the randomized phase III S0106 trial, evaluating GO versus no GO during induction and post-consolidation therapy in younger AML (17). Therefore, the drug was voluntary withdrawn from the US market in 2010 by the company, basing on an unfavorable safety-efficacy profile. More recently, we observed the rebirth of GO, with four different randomized studies demonstrating that the addition of GO to induction/consolidation improved EFS and OS in newly diagnosed AML patients, with a safe toxicity profile (17–20).

Moreover, when OS was analyzed according to cytogenetic profile, the beneficial effect of GO was documented most clearly in patients with favorable risk AML, followed by a smaller benefit intermediate-risk AML, but no benefit in adverse-risk. This was further highlighted in a recent meta-analysis of five large randomized trials, in which GO was combined with frontline induction/consolidation chemotherapy in both younger and older patients with AML (21). Remission rates were not increased, but by significantly reducing the risk of relapse, the overall survival at 5 years was improved irrespective of patient age (30.7% *vs* 34.6%). The survival benefit was particularly clear in those with favorable cytogenetics (55.2% *vs* 76.3%), but also observed in intermediate risk patients (34.1% *vs* 39.4%). Patients with adverse karyotype did not benefit on the overall analysis, nor individually within any of the five trials. Fractionated dosing using 3 mg/m^2^ were associated with less toxicity [myelosuppression and veno-occlusive disease (VOD)] with equal efficacy (21).

Hepatic VOD is a clinical syndrome characterized by jaundice and painful hepatomegaly and/or fluid retention, including ascites and encephalopathy as late manifestations. Initial studies with GO in AML demonstrated an increased risk of VOD, reported to occur after SCT in patients who received GO before SCT. GO is now reported in product labeling to pose risks for VOD based on clinical trial data. A wide range of biomarkers, including genetic, hematologic, hepatic, and inflammatory factors, as well as novel diagnostic techniques such as thromboelastography and measures of liver stiffness, may further enhance future risk calculation for VOD/SOS, although none of these has been widely adopted or studied prospectively in detail.

The example of GO showed how long and winding the road may be to develop and successfully commercialize a monoclonal antibody in AML. This is mainly due to two distinct issues: 1) the lack of an identified “ideal” antigen target in AML, which may result in the elimination of the chemo-resistant leukemic cells, and 2) the extra-medullary toxicities of monoclonal antibodies.

Preclinical studies suggest that GO would be most effective for AML with high CD33 expression (22), even in the absence of reproducible cut-offs among different laboratories (23). Although GO does not appear to benefit the non-CBF AML patients with the lowest CD33 expression, a higher GO dose may be more effective in CD33-low but not CD33-high younger adults (23). In the setting of elderly patients, unfit for intensive chemotherapy, receiving single-agent GO, low or minimal CD33 expression was associated with inferior outcome (24, 25). However, this issue remains controversial because other trials, particularly those in which GO was used in combination with chemotherapy, failed to identify a correlation between CD33 expression and clinical outcome (18, 19).

Given the vicissitudes of GO, several other antibodies targeting the CD33 antigen were developed in the last 10 years. Lintuzumab (HuM195) is a humanized monoclonal antibody that targets CD33, inducing antibody-dependent cell-mediated cytotoxicity and fixing human complement *in vitro* (26). The randomized phase III clinical trial of salvage chemotherapy with or without lintuzumab did not show any improvement in response rates or survival, in patients with refractory/relapsed AML (27). Other clinical trials with lintuzumab, alone or in combination with cytarabine in relapsed AML patients have been disappointing, mainly due to lack of efficacy (28, 29).

### Immune-Conjugated Targeting CD33

The anti-CD33 lintuzumab has been used as backbone for the development of immune-conjugated antibodies. To increase the antibodies potency without increasing the non-specific cytotoxicity associated with β-emitters, the α particle-emitting radionuclide bismuth-213 (213Bi) was conjugated to lintuzumab and evaluated in a phase I/II trial involving 31 patients with newly-diagnosed and relapsed/refractory AML, after partially cytoreductive chemotherapy. Myelosuppression lasting >35 days was dose-limiting; extramedullary toxicities were primarily limited to ≤grade 2 events, including infusion-related reactions. Transient grade 3/4 liver function abnormalities were seen in five patients (16%). Treatment-related deaths occurred in two of 21 patients (10%). The median response duration was 6 months (range, 2–12) (28).

In lintuzumab Ac225 the anti-CD33 antibody is conjugated with an alpha emitting isotope, Ac225. In the phase 1 trial enrolling elderly/unfit AML patients, lintuzumab Ac225 monotherapy was safely administered, with toxicity primarily limited to prolonged myelosuppression. As recently demonstrated in 15 RR/AML patients, the combination of lintuzumab Ac225 with the salvage chemotherapy regimen CLAG-M (cladribine, cytarabine, G-CSF, and mitoxantrone) resulted in 67% CR or complete remission with incomplete hematologic recovery (CRi) [with 100% overall response rate (ORR)], but was associated with prolonged myelosuppression. This regimen may represent a safe and potentially effective therapy for medically fit RR-AML pts, particularly as a bridge to allo-SCT (30, 31). Combining 0.5 µCi/kg lintuzumab-225Ac with venetoclax seems to preliminary have an acceptable safety profile, as reported in the first three patients enrolled in a phase 1 study recently presented at ASH 2020 (32).

SGN-CD33A is a humanized anti-CD33 antibody conjugated to a highly potent, synthetic DNA cross-linking pyrrolo-benzodiazepine dimer *via* a protease-cleavable linker. An interim analysis of a phase 1 dose escalation study of SGN-CD33A in patients with relapsed, CD33 positive, AML or those patients who declined intensive therapy, the composite complete remission (CRc) rate was 29%. Seventy-seven percent of patients who received doses of 40mcg/kg or higher had at least a 50% reduction in bone marrow blasts (33). A subsequent randomized phase III study evaluating azacitidine with or without SGN-CD33A (CASCADE study) was prematurely terminated due to increased early mortality in the SGN-CD33A arm, and this agent is no longer being developed in AML.

### Targeting CD123

CD123 is the α-chain of IL-3 receptor (34), overexpressed on stem/progenitor CD34+/CD38− AML cells (35, 36), associated with increased cycling activity, low CD34-high CD11b-highCD14 expression, increased cellularity at diagnosis, hypersensitivity to IL-3 stimulation and poor prognosis, as well as frequently associated with FLT3-ITD mutations (37–40).

Tagraxofusp (formerly SL-401) is an intravenously administered CD123-directed cytotoxin (composed of human interleukin-3 and a truncated diphtheria toxin payload) that was approved in December 2018 by the US FDA for the treatment of blastic plasmacytoid dendritic cell neoplasm (BPDCN). *In vitro*, tagraxofusp induced potent cytotoxic activity against CD123-positive AMLs and myelodysplastic syndrome blast cells (41). In preclinical models, tagraxofusp improved the survival of immunodeficient animals xenografted with primary AML cells (41).

The results of a phase I clinical trial performed on 45 AML patients showed that the maximum tolerated dose was 12.5 μg/Kg/day, with grade 2–3 adverse events including fever, hypoalbuminemia, transaminitis, hypotension and hypocalcemia, and low clinical responses (42). A phase I/II clinical trial NCT02270463 enrolled AML patients at risk of relapse who were not candidates for allo-SCT. Tagraxofusp was safe, but efficacy data are not yet available (43).

Resistance to tagraxofusp is mediated by downregulation of DPH1, the enzyme that converts histidine 715 on eEF2 to diphthamide, the direct target of ADP ribosylation by diphtheria toxin, an effect reverted by exposure to azacytidine (44), providing the preclinical rationale for the development of the combination of tagraxofusp with azacytidine in patients with R/R AML or in treatment-naive AML not eligible for standard induction therapy (NCT 03113643).

CSL360 is a recombinant, chimeric IgG1, anti-CD123 monoclonal antibody that neutralizes IL-3, with anti-leukemic activity *in vitro*. The phase 1 study enrolled 40 patients with advanced AML, and showed that doses ≥3.0 mg/kg resulted in complete saturation and down-regulation of CD123 and abolition of *ex vivo* proliferative responsiveness to IL-3, thus indicating an adequate blockade of IL-3 signaling. However, the trial failed to demonstrate anti-leukemic activity in most patients (45). To improve clinical efficacy, CSL362 (talacotuzumab), a fully humanized antibody, was developed, containing specific Fc-domain point modifications to enhance binding affinity with Fcγ receptors (46) and increase anti-leukemic activity *via* antibody-dependent cellular cytotoxicity (ADCC) against leukemic blasts and leukemic stem cells (47). In AML xenografts models in immune-deficient mice, CSL362 did not show any activity when administered as single agent, but was able to enhance the antileukemic effect induced by daunorubicin and cytarabine (47). The increased anti-leukemic activity *via* ADCC was confirmed by the first phase I clinical study in which CSL362 was tested in 40 R/R AML patients (NCT01632852) (48). A second phase I study (NCT01272145) enrolling high risk AML patients in first or second remission, who were not candidate for allo-SCT (45), showed preliminary activity of CSL362, with a relatively high conversion rate to negative minimal residual disease (49) or disease eradication in some patients (45). More recently, talacotuzumab was given to elderly, high-risk AML or MDS patients who have failed treatment with hypomethylating agents (50). Five of 24 AML patients achieved hematological improvement, providing the rationale to test the efficacy and safety of decitabine plus talacotuzumab versus decitabine alone in newly diagnosed AML patients ineligible for intensive chemotherapy (51). Unfortunately, the randomized multicenter study testing decitabine with or without talacotuzumab was terminated early due to lack of efficacy. As whole, immunotherapy based on naked anti-CD123 moAb appears to have limited benefits, probably due to the compromised immune profile of these patients (reduced mature NK cells, increased expression of inhibitory NK-cell receptors) (51).

IMGN632 is a CD123-targeting antibody-drug conjugate (ADC), in which a novel anti-CD123 antibody is coupled, *via* a peptide linker, to a unique DNA-alkylating cytotoxic payload of the recently developed IGN (indolinobenzodiazepine pseudodimer) class. IMGN632 reduced viability in AML cell lines and patient-derived samples, both *in vitro* and *in vivo* xenograft models, in the absence of or with limited myelosuppression (52). In a phase 1/2 clinical trial IMGN632 was administered as monotherapy to patients with CD123+ disease, including R/R AML and R/R BPDCN (53). 74 patients (67 AML, 7 BPDCN) received IMGN632 given in two schedules (dosing day 1 or fractionated dosing on days 1, 4, and 8) on a 21-day cycle. Seventy percent of patients were ELN adverse risk, 36% primary refractory to frontline therapy, and 19% of patients had received prior stem cell transplant. Of the seven R/R BPDCN patients, three (43%) achieved an objective response (CR, CRi, PR), and two had stable disease. Basing on these initial findings from the BPDCN cohort, IMGN 632 was granted an FDA breakthrough designation for BPDCN in November 16th, 2020. Of the 66 evaluable AML patients, 37 had a reduction in bone marrow blasts, and 13 (20%) achieved an objective response, comprising three CR and eighty CRi. IMGN632 was generally well tolerated. Severe neutropenia was the most common adverse event, and two reversible VOD cases occurred at dose levels ≥0.18 mg/kg, all in schedule A. As a whole, these data support further clinical exploration of IMGN362 in AML and it is currently being evaluated in various combinations with azacitidine and/or venetoclax alone or in combination, as well as an MRD-eradicating strategy (NCT04401748) (54).

Moreover, a phase I/II study recently presented at ASH 2020 found that IMGN632 was tolerable and resulted in a 29% ORR in patients with R/R BPDCN (8/28) with a CRc rate of 18% (5/28), and 31% ORR in patients with BPDCN previously treated with tagraxofusp (4/13) with a CRc of 15% (2/13) (55). IMGN632 is now being evaluated in both R/R and frontline BPDCN and appears to be an effective emerging therapy for BPDCN,

## T Cell Engagers

### Bispecific Antibodies Targeting CD33 and CD123

Another approach to immunotherapy in AML is retargeting the effector cells (T-cells, NK-cells, macrophages) to provide rapid activation, robust and durable cytotoxic responses, and potentially generate immunologic memory (56). The engagement of CD3 (part of the T-cell receptor) induces both proliferation of CD4 and CD8 T-cells and cytotoxic activity by CD8 and in part CD4 cells against the target, to eliminate cancer cells without genetic alteration of the T cells or need for *ex vivo* expansion/manipulation, providing off-the-shelf immuno-oncotherapy (56, 57).

In this scenario, the class of bispecific antibodies (bsAbs), also known as dual-targeting molecules, includes antibodies or single chain variable fragments (scFv) derived from the Fab fragment of the IgG immunoglobulin, composed of the VH and VL domains attached with a linker, to physically bridge two or more cells (58). Several formats of bsAbs have been investigated in AML: BiTe (Bispecific T-cell engager), BiKe (Bispecific killer cell engager), TriKe (Trispecific killer cell engager), Tandem Diabodies (TandAbs), and dual affinity retargeting antibodies (DARTs) (57, 59–61).

### BiTes and BiKes

In the BiTe and BiKe molecules, binding domains are two single-chain variable fragment (scFv) regions, arisen from mAbs, joined by a flexible peptide linker with affinities for both a selected leukemia associated antigen (e.g. CD33, CD19) as well as a selected target on an effector immune cell, like CD3 expressed on T-cells (in BiTes) or CD16 expressed on NK-cells (in BiKes) (62). Binding between effector cells and tumor cells facilitates the formation of cytolytic synapses inducing the apoptosis of the tumor cells and under specific circumstances the release of cytokines to amplify the immunological response by involving other immune cells or induce T cells proliferation (56–58, 63).

AMG330 is a human BiTe tandem single-chain antibody with the N-terminal specific for human CD33 and the C-terminal directed towards CD3Ԑ. *In vitro*, long-term cultures of primary AML blasts in the presence of AMG330 induced T cell activation, PDL1 over-expression on T-cells, release of IFN-γ, TNF-α, Interleukin-2, IL-10 and IL-6 (64, 65) and reduced bone marrow immune-suppressive CD14^+^ HLA-DR^low^ monocytic-like myeloid derived suppressor cells (66). These findings provided the rationale for combining AMG330 with pembrolizumab in an ongoing clinical trial (NCT04478695). Recently, a new approach based on the combination of T-cell engagers with immune checkpoint blockade in a single molecule, using a bifunctional checkpoint inhibitory T cell-engaging (CiTE) antibody that combines T-cell redirection to CD33 on AML cells with locally restricted immune checkpoint blockade has been proposed (67). *In vitro*, the PD-1 attachment increases T-cell activation (3.3-fold elevation of interferon-γ) and leads to efficient and highly selective cytotoxicity against CD33+PD-L1+ cell lines and patient-derived AML cells (n = 8). In a murine xenograft model, the CiTE induced complete AML eradication without infusion-related adverse events, as measured by body weight loss (67).

*Ex vivo*, AMG330 showed a potent dose- and effector to target cell ratio, dependent activity against human AML cell lines (64, 65). AMG 330 cytotoxicity was neither affected by common CD33 single nucleotide polymorphisms nor by expression of the adenosine triphosphate-binding cassette (ABC) transporter proteins, P-glycoprotein or breast cancer resistance proteins (68). Unlike bivalent CD33 antibodies, AMG 330 did not reduce surface CD33 expression (68). Moreover, AMG 330 resulted in significantly higher cytotoxicity in specimens from patients with newly diagnosed AML than those with relapsed/refractory disease despite similar levels of CD33 on myeloblasts (69).

*In vivo*, daily intravenous administration of AMG330 significantly prolonged the survival of immune-deficient mice adoptively transferred with human MOLM-13 AML cells and human T-cells (65). Preliminary data presented at ASH 2018 (70) of AMG 330 dosed up to 480 μg/d provided encouraging early evidence of tolerability and anti-leukemic activity in 40 heavily pre-treated patients with R/R AML (median prior therapies 4, range 1–5) (70). In the update presented at ASCO 2020, AMG 330 exhibited dose-dependent increase in steady state exposures over the studied dose range with clinical PK profile consistent with continuous IV administration. AMG 330 was administered to patients on an intra-patient step-up strategy of up to 480 µg, allowing the delivery of much higher doses of AMG 330 to the patients while lowering the risk for cytokine release syndrome (CRS). CRS is an emerging side effect of immune-therapy, manifesting as clinical syndrome characterized by a widespread immune inflammatory response due to the release of cytokines.

Among 42 heavily pretreated patients receiving a target dose ≥120 μg, there were eight responders (19%) including three CR, four CRi, and one morphological CR. The median response duration was 40 days (range: 14–121). Responding patients were allowed to proceed to allo-SCT. There were more responders among patients with a lower (<25%) leukemic burden at the time of treatment initiation, and there was no correlation between response and CD33 expression on leukemic blasts (71).

AMG673 is a new anti-CD33x anti-CD3 BiTE antibody construct, that binds both CD33 and CD3 and is genetically fused to the N-terminus of a single-chain IgG Fc region, thereby potentially increasing the half-life to 7 days which permits weekly dosing of the agent. In the phase 1 trial (NCT03224819), AMG 673 was administered as two, short, and intermittent iv infusions during a 14-day cycle in adult patients with R/R AML. Preliminary data presented at ASH 2019, on the first 30 patients, receiving AMG 673 dosed up to 72 µg, provided early evidence of the molecule’s acceptable safety profile and anti-leukemic activity. Assessment of bone marrow in treated patients showed a decrease in blasts in 12/27 (44%) evaluable patients, of which six experienced ≥50% reduction in marrow blasts compared with baseline. One patient achieved CRi with 85% reduction in bone marrow blasts at a dose of 36 µg (72).

G333 is single chain bispecific anti-CD3 × CD33 antibodies arranged in a tandem format using a novel linker for redirecting of T cells to AML blasts (73), without effect on normal human CD34+ hematopoietic stem and progenitor cells in both colony forming assays or in NSG repopulating experiments (74). Currently, G333 is tested in the ongoing clinical trial NCTT03516760. No data are available up to now.

### Tandem Diabodies

Tandem diabodies combine two scFVs for each target connected with a single polypeptide target. This allows them to maintain the avidity of a bivalent antibody as well as have a molecular weight that exceeds the renal clearance threshold (58).

AMV564 is a tetravalent anti-CD33 × anti-CD3 TandAb with a 106 kDa molecular weight avoiding first pass renal clearance and resulting in a longer half-life in comparison to BiTEs. Preclinical data with cryopreserved human AML specimens demonstrated *in vitro* activity across the spectrum of disease stage, cytogenetic risk and CD33 expression levels, leading to the first in human phase I trial in patients with RR/AML (NCT03144245). Preliminary results presented at the EHA in 2018 showed evidence of T-cell activation by increased cytokine levels and expression of antigen markers of T-cell activation. There was initial evidence of activity, with 13–38% reductions in the bone marrow blasts in 10/16 patients. The treatment was generally well tolerated with no grade 3–4 toxicities attributed to the study treatment with manageable grade 2 CRS observed at the 50 mcg/day dose in only one patient.

### Dual Affinity Retargeting Antibodies

Dual affinity retargeting antibodies (DARTs) consist of variable domains of two antigen-binding specificities linked to two independent polypeptide chains. Each variable domain is formed by associating one VL segment on one chain with a VH segment on a second chain, covalently linked *via* disulfide bridge (75).

The first-in-class DART with clinical activity in AML is flotetuzumab (MGD006 or S80880), based on two independent polypeptides fusing the heavy-chain variable domain of one antibody to the light-chain variable domain of the other to connect CD3 and CD123. *In vitro* studies demonstrated a dose-dependent killing of AML cells (76). *In vivo*, the infusion of the bispecific antibody into immunodeficient mice xenografted with AML blasts resulted in total clearance of peripheral blood leukemic cells and subtotal elimination of leukemic cells in bone marrow (77), providing rationale for the first phase I/II trial (78, 79). In advanced AML/MDS patients, flotetuzumab showed an acceptable safety profile, and was able to induce a CR/CRi rate of 31% in patients with primary refractory disease, but surprisingly no responses in relapsed patients (0/11) (80).

Recently, a large multicenter, open-label, phase 1/2 study of flotetuzumab enrolled 88 adult R/R AML patients (80). 42 in the dose-finding and 46 at the recommended phase 2 dose (RP2D) of 500 ng/kg/day. Among 30 patients with primary induction failure (PIF) or early relapse (ER) treated at the RP2D, the CR/CRh rate was 26.7%, with an overall response rate (CR/CRh/CRi) of 30.0%. In patients achieving CR or CR with partial hematological recovery (CRh), median OS was 10.2 months with 12-month survival rate of 50%. Interestingly, Bone marrow transcriptomic analysis showed that a parsimonious 10-gene signature predicted complete responses to flotetuzumab (AUROC = 0.904 versus 0.672 for the ELN risk classifier). Moreover, flotetuzumab induced CR in almost half of TP53 mutated patients enrolled. Patients with TP53 abnormalities who achieved a complete response experienced encouraging OS (median 10.3 months; range, 3.3–21.3 months) (81).

Given the interesting data on patients with PIF/ER, an update was recently given at ASH 2020 (82). Twenty-four PIF and 14 ER AML patients were treated at the RP2D (median age 63 yrs); the large majority (94.7) had non-favorable risk by ELN 2017 criteria. CRS was the most common side effect, with all patients experiencing mild-to-moderate (grade ≤2) CRS. The incidence of CRS progressively decreased during dosing at RP2D, allowing outpatient treatment in most cases. The overall CR/CRh/CRi rate was 42.1%, with 68.8% of patients subsequently able to undergo allo-SCT. Median OS was 4.5 months, with a median follow up time of 10.8 months, in the entire population, and 7.7 months in responding patients (82). These results show encouraging activity in a population with poor prognosis and high unmet medical need.

A next-generation CD3-engaging Fc-bearing DART platform was generated through CD3 arm affinity modulation. These DART variants feature extended PK and an expanded TI suitable for potential clinical development in hematological malignancies, with promising preliminary results (83).

Vibecotamab (XmAb14045) is a bispecific monoclonal antibody, with a full-length immunoglobulin molecule requiring intermittent infusions, targeting both CD123 and CD3 and stimulating targeted T cell-mediated killing of CD123-expressing cells. In the update presented at ASH 2020, 104 patients with AML, were treated at dosages from 0.003 to 12.0 µg/kg. The recommended initial priming dose is 0.75 µg/kg; no MTD has been identified. CRS was the most common adverse event, occurring in 62 of 106 patients (85% grade 1–2, 15% grade ≥3), mostly with the first dose. No myelosuppression requiring dose modification or evidence of tumor lysis syndrome was reported. Overall response rate was 14% [2 CR, 3 CRi 2 morphologic leukemia-free state (MLFS)] occurring in patients treated at higher doses, mostly after the first cycle of therapy (0.75 µg/kg). Stable disease (SD) was observed in an additional 36 patients (71%). Characterization of responders versus non-responders revealed responding patients harbored a lower pretherapy burden of disease and specific T-cell subtypes. No association was found between response status and CD123 target expression on AML blasts (84).

Recently, the development of a dual-targeting triple body 33-16-123 (SPM-2) agent, with binding sites for target antigens CD33 and CD123, and for CD16 to engage NK as cytolytic effectors was reported, with promising clinical activity. Blasts from all 29 patients, including patients with genomic alterations associated with an unfavorable genetic subtype, were lysed at nanomolar concentrations of SPM-2. Maximum susceptibility was observed for cells with a combined density of CD33 and CD123 above 10,000 copies/cell. Cell populations enriched for AML-LSCs (CD34pos and CD34pos CD38neg cells) from two AML patients carried an increased combined antigen density and were lysed at correspondingly lower concentrations of SPM-2 than unsorted blasts. These initial findings raise the expectation that SPM-2 may also be capable of eliminating AML-LSCs and thus prolong survival (85).

## Adoptive T Cell Therapies

In the last years, several attempts have been made to use effector immune cells, namely T and NK cells, as a means of adoptive immunotherapy against AML. Regarding T cells, despite important limitations mostly related to the absence of a dispensable antigen, recent advances have been made to develop safe and effective chimeric antigen receptor (CAR) T cells redirected against myeloid antigens to be used for the treatment of patients with AML. As for NK cells, different approaches, i.e. freshly isolated, *in vitro* expanded and/or activated, have been widely investigated at the preclinical level with important and promising early clinical results. Here we summarize the most relevant evidence supporting the translational use of CAR T cells and NK cells as a mean of adoptive immunotherapy against AML (**Table 2**).

**Table 2:** Adoptive cell therapies.

Category	Drug name	Drug details	Antigen/target	Combination therapy	Indication (AML only)	Effects *in vitro o vivo*	Refs
**CAR-T cell therapy**	CD123 CAR-T	Second generation CAR-T	CD123	Alone	R/R AML	Beneficial effect but data not available	(76, 85–93)
	CD33 CAR-T		CD33	Transplantation of stem/progenitor cells engineered to ablate CD33	R/R AML	Data not available	(94, 95)
	CLL CAR-T	CD33-CLL1 dual specific CAR-T cells	CEC12A or CLL1	All patients received conditioning of fludarabine and cyclophosphamide	R/R AML	High efficacy and manageable toxicity	(102–104)
**NK cell therapy**	Haploidentical NK cells	KIR-mismatch NK cells	KIR-KIR-L	Haploidentical NK cells	Advanced AML; high risk AML in CR	Prolonged EFS	(108–115)
	IL-2, IL-15	Interleukin	NK cells	Alone	R/R AML	NK-cell expansion; improved CR	(119–122)
	Prime haploidentical NK cells	Prime haploidentical NK cells activated with IL-2	KIR-KIR-L	Enriched with PBMC (T-and B-depleted)	R/R AML	High efficacy, favorable safety profile	(123)
	CNDO-109-NK cells	Leukemia cell line lysate (CNDO)-activated donor-derived haploidentical NK cells	KIR-KIR-L	As consolidation therapy	High risk AML in CR	High efficacy, favorable safety profile	(124, 125)
	Expanded NK cells	Expanded NK cells *in vivo* by K562 cells expressing IL-21	KIR-KIR-L	Before and after haploidentical SCT	High risk AML	High efficacy, favorable safety profile	(126–134)

### CAR-T Cells

CARs are engineered cell surface molecules that link a target-cell ligand-recognition domain to signaling regions from the T-cell receptor (TCR). CARs are synthetic molecules resulting from the fusion of an extracellular antigen-binding domain and intracellular signaling domains, capable of activating T cells. In CAR design the recognition domain is of foremost relevance and, ideally, the antigen chosen as the CAR target should be mostly expressed on tumor cells while being absent or weakly expressed on the normal cellular counterparts. In the setting of AML this is particularly challenging, as the expression of potential targets mostly overlap on cells within normal myeloid cell compartment (76, 86), resulting in high toxicity and myelosuppression. To now, this issue has represented the major limitation in the development of CAR technology for AML immunotherapy.

In 2013 Ritchie et al. reported the first clinical experience of CART therapy in AML utilizing a second generation CAR directed against the Lewis Y antigen, demonstrating some preliminary activity and, most importantly, the absence of overt hematological toxicity (87). Different antigens have been tested as potential target for CAR-T cell development in AML.

### CD123 CAR-T Cells

CD123 is highly and ubiquitously expressed on AML cells, but it is also detectable on normal HSPCs (88). Despite these limitations, CD123 is an attractive target, as proven by the great number of preclinical and early clinical studies, addressing this antigen for AML immunotherapy. Indeed, anti-CD123 monoclonal antibodies and CD123 CAR-T cells are currently under investigation and in early clinical development. CD123 CAR-T cells were preliminarily tested in preclinical models. In mice a first-generation anti-CD123 CAR was to modify human T cells, which demonstrated a potent effector activity against cell-line and primary AML cells, leading to long term survival of mice and to the establishment of a T-cell memory pool able to reject disease (89).

A second generation of CARs was, then, developed, containing a CD123-specific single-chain variable fragment, in combination with a CD28 costimulatory domain and CD3ζ-signaling domain and targeting different epitopes on CD123 (90), even if a recent study suggest that there is no evidence that normal HPCs express different splice variants than AML blasts (91). These second generation CD123 CAR-T cells activated T-cell effector functions against poor-risk primary AML patient samples and were able to lyse autologous AML blasts *in vitro*. Based on these premises, phase 1 clinical trials are currently on-going.

Patients with R/R AML received CD123-specific CAR T cells, obtained *via* mRNA electroporation rather than by using lentiviral transduction, after lymphodepleting chemotherapy (92). No significant clinical responses were observed, but a certain degree of activity exerted by CART cells was documented as demonstrated by the CRS occurring in all treated patients. Of interest, the use of mRNA electroporation resulted in transient CAR T cell detection *in vivo*, thus reducing myelosuppression and extra-hematologic toxicity. Whether such a reduced persistence, which certainly provides a favorable safety profile, may negatively impact on the efficacy of the whole approach will be the question to be answered in the open and ongoing trial at the University of Pennsylvania (NCT03766126).

Another trial is on-going at the City of Hope Medical Center, employing a second generation CD123-specific CAR construct to transduce T cells (NCT02159495). Different doses have been used. Twenty-four patients with refractory AML have been enrolled. Interestingly, some clinical responses in patients receiving the highest dose have been described, including in few cases the achievement of complete remission, which allowed to proceed to allo-SCT (93).

### CD33 CAR-T Cells

CD33 is ubiquitously expressed on AML cells, but its expression on normal myeloid cells makes it a non-ideal target for a CAR-based therapy. Some case reports indicated some responses, but very few mature clinical data are available. A Chinese phase I clinical trial have studied the feasibility of anti-CD33 CAR in the treatment of relapsed or refractory AML. Only one patient was treated. Suggestions of a beneficial effect were present, but severe side effects as fever, CRS and pancytopenia were reported (94).

Recently, an approach to treat AML by targeting the CD33 has been proposed, combining CD33-targeted CAR-T cells with the transplantation of hematopoietic stem cells that have been engineered to ablate CD33 by using CRISPR/Cas9 technology in human stem/progenitor cells (HSPC). Of note, CD33-deleted HSPCs maintain their ability to engraft and to repopulate normal haematopoiesis. In a mouse xenograft model the transplantation of CD33-ablated HSPCs with CD33-targeted immunotherapy leads to leukemia clearance, without myelosuppression. A clinical trial evaluating safety and toxicity of such approach is currently under design for R/R AML patients at the University of Pennsylvania (95).

Cytokine-induced killer (CIK) cells may be considered a third way in cancer immunotherapy representing a terminally differentiated CD3^+^ CD56^+^ T cell population with both T-cell and NK cell-like phenotype and functionality. Encouraging results in initial clinical studies have been achieved in cancer patients treated with CIK cells (96). A description of CIK cells is out of the scope of this review. However, it is notable that in the AML setting CD33 has been recently tested as target for preclinical validation of a novel strategy with CAR engineered CIK cells as a means of adoptive immunotherapy (97).CIK cells have been transduced with a CD33.CAR by using a non-viral system, which offers improved transfection efficiency. CD33.CAR-CIK cells showed significant antileukemic activity *in vitro* and, when used in patient-derived AML xenograft models, were capable of contrasting AML development both as early treatment and in mice with established disease. These data may support further development and implementation of CD33 CAR-CIK cells into early clinical trials.

### CD44v6 CAR-T Cells

The adhesive receptor CD44 is a glycoprotein involved in cell-cell and cell-matrix interactions and is widely expressed both in hematological and solid neoplasms. Its isoform containing the variant domain 6 of CD44 gene (CD44v6) is involved in several aspects of tumorigenesis and contributes to the cancer stem cell phenotype (98). In the setting of hematological malignancies, Casucci et al. provided the first evidence that silencing of CD44v6 may prevent engraftment of AML cells in mouse models (99). Accordingly, CD44v6-CAR-T cells have been demonstrated to mediate potent antitumor effects against primary AML and MM while sparing normal hematopoietic stem cells. The co-expression of a suicide gene enabled fast and efficient pharmacologic ablation of CD44v6-targeted T cells, thus preventing hyperacute xenogeneic graft-versus-host disease. Based on these promising clinical results, CD44v6-targeted CAR-T cells in AML and MM are being explored in a Phase I/II clinical trial, to evaluate their safety and early efficacy (NCT04097301).

### FLT-3 CAR-T Cells

FMS-like tyrosine kinase 3 (FLT3) is a transmembrane receptor tyrosine kinase involved in crucial function of normal of hematopoietic stem/progenitor cells, such as survival, proliferation, and differentiation. In AML, FLT3 is mutated in approximately one-third of patients, resulting in increased survival of leukemic cells and conferring poor prognosis. Recent preclinical data have investigated the targeting of FLT3 by means of adoptive immunotherapy with specific CAR-T cells Since FLT3 is also expressed on normal HSCs and on early hematopoietic progenitors (HPs), besides its anti-leukemic activity, the safety profile of anti-FLT3 CAR-T is of the utmost importance. CD8^+^ and CD4^+^ T-cells expressing a FLT3-specific CAR were engineered, demonstrating potent reactivity against wild-type or mutated FLT3 AML cells (100). Of interest, the FLT3-inhibitor crenolanib increased surface expression of FLT3 thus eliciting CAR-T cells recognition and activity. However, FLT3-CAR T-cells also recognized normal HSCs *in vitro* and *in vivo*, being profoundly myelotoxic. To overcome some of these drawbacks related to the targeting of FLT3, one recent study reported the preclinical evaluation of an off-the-shelf CAR T cell therapy targeting FLT3 for the treatment of AML with reduced myelotoxicity while sparing anti-leukemic activity. Indeed, by using a surrogate CAR with affinity to murine FLT3, rituximab-mediated depletion of FLT3 CAR T cells after AML eradication enables bone marrow recovery without compromising leukemia remission (101).

### CLL1 CAR-T Cells

Also known as CEC12A, CLL1 (C-type Lectin-like Molecule 1) is highly expressed in AML cells. Although some reports indicate its absence on normal HSPCs, some others indicate that it may be detectable on monocytes and immature early progenitors. Interestingly, non-hematological cells lack CLL1 expression (102). Preclinical studies demonstrated the specific activity of a CLL-1-specific chimeric antigen receptor T cells (CLL-1 CAR-Ts) against CLL-1^+^ AML cell lines and primary AML patient samples *in vitro*. Moreover, in a human xenograft mouse model, CLL-1 CAR-Ts mediated anti-leukemic activity against AML prolonging survival. Of note, the colony formation of normal progenitor cells remained intact following CLL-1 CAR-T treatment, suggesting a differential sensitivity of leukemic versus normal myeloid compartment to the cytotoxic effect of CLL-1 CAR T cells (103).

Based on this preclinical evidence, some very preliminary clinical data have been recently reported. CD33-CLL-1 dual specific CAR T cells have been used in a first-in-human clinical trial, based on the observation that AML samples expressing CD33 are mostly CLL-1-positive. Seven of nine patients were MRD^-^ by flow cytometry, two of nine had no response, one of which was CD33^+^/CLL1^−^, indicating the importance of CLL1 target in the CAR-T treatment. For the seven patients who reached MRD-negativity, six patients moved to a subsequent allo-SCT (104).

To overcome the major limitations of CAR T cell therapy in AML, several strategies are currently under active investigation. The attempt to create an artificial “*AML-specific*” antigen by genetic editing of allograft has been mentioned before about CD33. Another strategy aims at limiting CAR T cell persistence to prevent myeloablation by using a kind of “safety switch” incorporated in transgenic T cells. Indeed, suicide genes, such as herpes simplex virus-thymidine kinase (HSV-tk), which allows the selective elimination of expressing cells, have been widely tested in different immunotherapy settings. Recently, it has been evaluated in the setting of CAR-T cells in preclinical studies (91, 105).

Beyond the identification of an optimal target antigen and of the best strategy to prevent prolonged and not acceptable myelosuppression, a very active area of preclinical and biological research points toward the identification of the immunosuppressive effects induced by AML microenvironment in dampening the therapeutic response of adoptively transferred CAR-T cells, similarly to what observed in other settings of adoptive immunotherapy. In this context, the expression and function of immune checkpoint receptors on AML blasts as well as other immunosuppressants are being tested.

## Adoptive NK Cells Therapies

Natural killer (NK) cells are lymphocytes belonging to the innate immune system. Their cytotoxic activity is tightly regulated by the balanced expression of a wide and complicated pattern of activating and inhibitory receptors, among which inhibitory receptors called killer-immunoglobulin like receptors (KIRs) have been extensively characterized (106). In cancer immunology, NK cells have been shown to mediate antitumor immunosurveillance both in solid tumors and hematological malignancies, including AML (107). Indeed, NK cells are potent effectors which can target and kill leukemia cells without prior exposure to those cells (108). Based on their crucial role, several strategies of NK cell-based immunotherapy are currently under active preclinical and clinical investigation and early data from clinical studies have been reported (76). A wide variety of sources of therapeutic NK cells have been proposed both at preclinical and early clinical level, including haploidentical NK cells, umbilical cord blood NK cells, stem cell-derived NK cells, NK cell lines, adaptive NK cells, cytokine-induced memory-like NK cells and CAR NK cells (109). According to the main scope of this review, herein we intend to summarize those strategies that are being investigated in the field of AML with a special focus on those entered into the clinical ground.

### Infusion of Haploidentical NK Cells

NK cells have a major role in the eradication of residual AML cells after haploidentical SCT (108), boosting *graft-versus-leukemia* (GVL) effects without exacerbating *graft-versus-host* disease (GVHD). In the setting of haploidentical transplantation, the KIR-KIR-L mismatch in the graft versus leukemia direction, which defines the donor’s NK alloreactivity, has been shown to significantly impact on the post-transplant clinical outcome in virtue of its effect in regulating donor NK cell cytotoxicity against AML cells (108, 110, 111).

Based on these premises, several groups have sought to translate this notion to the non-transplantation setting by using infusion of haploidentical NK cells as a means of adoptive immunotherapy for patients with advanced AML. Outside the transplant setting, in their pivotal study Miller and colleagues (112) first demonstrated that adoptive immunotherapy with haploidentical NK cells in AML patients was feasible and safe and was effective in controlling leukemia relapse. In a retrospective analysis, the authors also showed that a better response was associated with KIR-L mismatch between donor and recipient.

Consequently, adoptive immunotherapy with haploidentical KIR-mismatched NK cells has become an area of translational research for AML patients. Several reports demonstrated the feasibility and early clinical efficacy of selecting and infusing highly purified, T cell-depleted, KIR-mismatched NK cells to consolidate remission in high-risk patients with AML (113–115). Of note, the percentage of donor-derived alloreactive NK cell clones before infusion was significantly correlated with relapse occurrence, indicating that a functional dose of 2 × 10^5^/kg alloreactive NK cells may be predictive of response (115). Based on these data, a multicentre clinical trial of adoptive immunotherapy with haplodentical KIR-L mismatched alloreactive NK cells to consolidate remission in AML patients not eligible for allo-SCT is currently on-going **(NCT03955848).** By using a “donor versus no donor” approach, patients receiving NK cell infusion will be compared to a control arm, receiving standard-of-care. Efficacy in terms of relapse rate and overall survival is the primary objective of the trial.

### NK Cell Activation

To potentiate effector cell function of infused NK cells several approaches, both *in vivo* and *in vitro*, of NK cell activation have been tested. To this end, *in vivo* interleukin (IL)-2 administration has been widely used, resulting in expansion and cytotoxicity enhancement of infused NK cells (112, 114, 115). However, IL-2 is well-known to increase the number and the suppressive function of regulatory T cells (T_regs_) (109), which in turn antagonize NK cells by reducing their expansion capacity and effector function (116–119). Accordingly, the number of circulating T_regs_) after NK cell infusion critically influences the capacity of infused NK cells to expand and to kill AML cells (120). Of note, depletion of host T_regs_ by an IL-2 diphtheria toxin fusion protein was associated with increased NK cell expansion and higher response rates in adults with relapsed AML, resulting in better overall and disease-free survival (120).

To overcome some of the limitations associated with IL-2 administration, other cytokines are currently under investigation for *in vivo* activation of NK cells. In particular, IL-15 stimulates both NK cells and T cells. IL-15 in complex with its IL-15R-a receptor induces a potent proliferative signal that expands NK-cell and CD8 T-cell populations in murine models (121).

Recently, the results of two first-in-human phase 1 and phase 2 trials of intra venous and subcutaneous rhIL-15 given with haplo-NK-cell therapy after lymphodepletion to treat R/R AML have been reported (122). Forty-two patients received either intravenous (IV) or subcutaneous (SC) recombinant human IL-15 (rhIL-15). Robust *in vivo* NK-cell expansion at day 14 was observed in a significant fraction of patients and correlated with the achievement of some complete responses. Of note, rhIL-15 induced better rates of *in vivo* NK-cell expansion and remission compared with previous trials with IL-2, but it was associated with previously unreported CRS after subcutaneous but not intra-venous dosing.

Activation of NK cells to be used as a means of adoptive immunotherapy may be also obtained by priming NK cells before infusion. *Ex vivo* activation may have the advantage to avoid some of the detrimental and toxic effects associated with *in vivo* cytokines administration. To this aim, overnight activation with IL-2 was used to prime haploidentical NK cells before infusion in a cohort of R/R AML and high risk-MDS patients (123). NK cells were enriched from non-mobilized donor peripheral blood mononuclear cells (PBMC) after T- and B-cell depletion. The number of responses, including complete remission, was promising, allowing in some cases to proceed to allo-SCT. Biological studies were performed, suggesting a correlation between clinical response and the frequency of donor NK cells at day 7/14 as well as reduced activation of CD8 T cells and lower levels of inflammatory cytokines after infusion (123).

Human NK cells primed with the CTV-1 leukemia cell line lysate CNDO-109 have been shown to exhibit enhanced cytotoxicity against NK-cell-resistant cell lines (124). On this basis, a phase 1 trial with CNDO-109-activated donor-derived haploidentical NK cells (CNDO-109-NK cells) was conducted in AML patients who had obtained CR at high risk for relapse as part of a consolidation strategy (125). Before CNDO-109-NK cell administration, patients were treated with lymphodepleting fludarabine/cyclophosphamide. CNDO-109-NK cells were well tolerated and durable CRs were observed in some cases, which may suggest a clinical activity of the whole strategy warranting further studies.

### *Ex Vivo* Expansion of NK Cells

In the development of NK cell-based adoptive immunotherapy, one major limitation has been the collection of a sufficient number of NK cells to reach a therapeutic effect. For this reason, several attempts to expand NK cells have been made both at preclinical and clinical level. Most studies have used cytokines, such as IL-2 and/or IL-15, as a means of *ex vivo* NK cell expansion (126–128).

Recently, other approaches have been tested combining cytokine-based priming with additional stimuli. One method to expand NK cells *ex vivo* using K562 feeder cells expressing membrane-bound IL-21 (mbIL21).25 has been recently reported. This approach expands highly functional NK cells up to 35,000-fold in 3 weeks (129), providing the preclinical rationale for a phase 1 study to determine safety, feasibility, and maximum tolerated dose (130). Thirteen patients with myeloid malignancies, including AML, at high risk of disease occurrence were treated before and after haploidentical SCT to prevent relapse. Lymphodepleting chemotherapy was administered before infusion. No infusion reactions or dose-limiting toxicities occurred. Of interest, NK-cell reconstitution was superior to that observed in a similar group of patients not receiving NK cells. Clinically, infusion of NK cells was associated with improved low relapse and incidence of viral infections. To achieve high number of functional NK cells, NK-cell product for adoptive immunotherapy was also generated *ex vivo* from CD34+ hematopoietic stem and progenitor cells (HSPC) which had been obtained from partially HLA-matched umbilical cord blood units (131). Moving from the hypothesis that generating more homogeneous and well-defined allogeneic NK-cell products may be associated with better clinical results, the authors had previously developed a culture system where CD34+ HSPC isolated from allogeneic umbilical cord blood (UCB) were expanded and differentiated into NK cells in the presence of IL15 and IL2, resulting in a clinically relevant dose of highly purified NK cells (132, 133).

These allogeneic HSPC-NK cells showed cytolytic activity against AML cells *in vitro* and *in vivo* mouse models (134). Ten AML patients who had achieved complete remission received escalating doses of HSPC-NK cells after lymphodepleting chemotherapy. *In vivo* cytokine administration was not allowed. Infusion was well- tolerated, and the toxicity profile was favourable. Of interest, despite the absence of *in vivo* cytokine boosting, HSPC-NK cells were clearly detectable in a significant fraction of patients. Donor chimerism was documented also in the BM of infused patients. From a clinical point of view, it is noteworthy that some patients harbouring minimal residual disease (MRD) in bone marrow became MRD negative. These findings suggest that HSPC-NK cell adoptive transfer is a promising and, importantly, potential “*off-the-shelf*” strategy for NK cell-based adoptive immunotherapy in AML.

## Checkpoint Blockade and Macrophage Checkpoint Blockade

Immune checkpoint inhibitors (ICPI) work by blocking checkpoint proteins from binding with their partner proteins. This prevents the “off” signal from being sent, allowing the T cells to kill cancer cells. PD-1 is a co-inhibitory molecule expressed on T cells, B cells, and myeloid cells. Binding of PD-1 to the B7 family of ligands such as PD-L1 on tumor cells results in suppression of proliferation and immune response of T cells, also in AML (135, 136). Indeed, in newly diagnosed and relapsed AML patients, PD1 expression is higher than healthy controls, and can mediate immune-evasion of leukemic blasts (77, 137). PD-L1 overexpression in AML blasts is associated with inferior clinical outcome in patients with mutated nucleophosmin and concomitant internal tandem duplications in the FLT3 gene (138), and positively correlated with poor risk cytogenetic and TP53 expression (139). Moreover, PD-1 over-expression on lymphocytes as well as its two ligands PD-L1 and PD-L2 on CD34+ AML cells, may contribute to treatment resistance to azacytidine (137), providing the rationale to combine hypomethylating agents (HMA) and ICPIs in the treatment of AML (76, 140, 141).

In a pivotal phase 1/1b trial, involving 28 patients with relapsed hematologic cancer after allo-SCT, ipilimumab was feasible, although immune-mediated toxic effects and GVHD occurred. Durable complete responses were observed in five of 14 patients treated with the higher dose of Ipilimumab (10 mg/kg), including patients with extramedullary AML. Responses were associated with *in situ* infiltration of cytotoxic CD8+ T cells, decreased activation of regulatory T cells, and expansion of subpopulations of effector T cells in the peripheral blood (142).

Thus, PD-1 inhibitor nivolumab has been evaluated as single-agent maintenance in AML patients with high-risk features including adverse cytogenetics, high risk mutations, secondary/therapy-related AML or AML with residual MRD after induction-consolidation (143) with promising preliminary results. Grade 3–4 immune-related adverse events occurred in five patients, one patient had autoimmune hemolytic anemia and came off study. With a median follow-up of 19.3 months, the median OS was not reached and the median CR duration was 8.3 months in 14 patients (median age, 56 years). After a median follow‐up of 11 months (range, 1.4–26 months) the 6‐ and 12‐month CR rates were 79 and 71%, respectively, and 12‐ and 18‐month OS rates were 86 and 67%, respectively; the regimen was well tolerated (143). Controversies are emerging in the setting of post–allo-SCT relapse, with limited anti-tumour efficacy due to multiple adverse events, requiring dose de-escalation (144).

The ongoing randomized phase 2 trial (REMAIN) is comparing surveillance and single‐agent therapy with nivolumab for AML in patients, post consolidation, who are not candidates for HCT (NCT02275533). The final results are expected in late 2021.

Another phase IB/II study tested nivolumab in combination with azacitidine in 70 relapsed AML (NCT02397720), resulting in 33% ORR. The combination was well tolerated with 11% grade 3–4 immune related adverse events (145, 146). The combination produced an encouraging response rate and OS, especially in HMA-naïve (overall response rate >50%) and salvage1 patients (median OS 10.5 months), respectively (146). Immune mediated Grade 2-4 toxicities were seen in 22% of the patients and involved lungs, renal, skin, GI and endocrine systems. Immune profiling using flow-cytometry and mass cytometry demonstrated that patients with increased CD3 or CD8 infiltrate in the bone marrow pretherapy were more likely to respond, suggesting that marrow T-cell infiltration may be a useful biomarker that could be used to prospectively identify patients most likely to benefit from PD1 based therapy in the future.

Pembrolizumab is another PD-1 inhibitor tested in R/R AML setting. In 37 RR patients, high dose cytarabine (<60 years: 2 gm/m2 IV Q12hours days 1–5; >60 years: 1.5 gm/m2 IV Q12hours days 1–5) was followed by pembrolizumab 200 mg IV on day 14, resulting in 38% CR/CRi leading to 24% patients proceeding to allo-SCT. There were no instances of Grade >3 acute GVHD or veno-occlusive disease post-allo-SCT, and immune-related adverse events (iRAEs) were uncommon (Grades 2–4 iRAE 13%) despite administration after cytotoxic chemotherapy. Median follow-up among survivors, and median OS, EFS and DFS was 7.8, 8.9, 6.9, and 5.7 months, respectively (147).

In another study, Pembrolizumab 200 mg beginning on D8, and every 3 weeks thereafter, was tested in combination with azacytidine 75 mg/m^2^ days 1–7 in both R/R and newly diagnosed older AML (148). Among 29 evaluable R/R patients, CR/CRi and PR rate were 14 and 4%, respectively. Moreover, four additional patients experienced hematologic improvement (HI) (14%), and seven (24%) stable disease (SD) for at least four cycles. The median EFS was 6 months, and median DFS for CR patients was 8.5 months. In the elderly, frontline setting, among 17 evaluable patients, eight achieved CR/CRi (6/2) (47%), two PR (12%), 2 HI (12%), and four SD for at least six cycles (24%). With a median follow-up of 19 months, the median OS was 13.1 months. Intriguingly, the median DFS for CR/CRi patients was 16.6 months, showing a relevant clinical activity for the combination in elderly AML patients (148).

The addition of nivolumab to frontline therapy with idarubicin and cytarabine has been evaluated in 44 patients with newly diagnosed AML or high-risk MDS in a phase 1/2 study (149). At a median follow-up of 17 months, median event-free survival was not reached, and the median OS was 18 months; 43% patients achieved a response and proceeded to allo-SCT, with grades 3–4 GVHD observed in 26%, providing feasibility and efficacy of addition of nivolumab to induction chemotherapy with idarubicin and cytarabine (149).

A randomized phase III and a randomized phase II study of azacitidine with or without PD-1 inhibitor in frontline elderly AML (NCT03092674, NCT02775903) and a randomized trial of PD-1 inhibitor for eradication of MRD in high-risk AML in remission (NCT02275533) have been initiated. Clinical and immune biomarker enriched trials are likely to yield further improved outcomes with HMA+ICPI therapies in AML and are strongly encouraged. Furthermore, recent data suggest that patients who receive ICPI based therapies for AML or MDS are able to safely proceed to allo-SCT without significant increase in risk of post SCT GVHD, acute febrile episodes or veno-occlusive disease (146). The use of post allo-SCT Cytoxan seems to be significantly beneficial in this specific setting.

CTLA-4 blockade with ipilimumab (IPI) was not effective as single agent in patients with hematological malignancies relapsing after allo-SCT (142, 150). Recently, IPI was investigated in association with decitabine a phase I, multicenter, investigator-initiated study (CTEP 10026) in patients with R/R MDS/AML with (Arm A) and without (Arm B) prior allo-SCT. Safety profile was favorable, with approximately 50% of patients developing grade 1–2 IRAEs, comprising late-onset acute (grade III, colon/liver, one patient) or chronic (four patients, one severe) GHVD. All IRAEs were managed though steroids administration, except for the grade III acute GVHD, steroid refractory, complicated by fatal septic shock. Objective responses were detected in eight of 16 evaluable patients (three CR, two CRi, and three marrow CR), resulting in a median OS of 18.3 months (95% CI: 11.7-NA) (151).

T-cell immunoglobulin and mucin domain 3 (Tim-3) is a checkpoint receptor expressed by a wide variety of immune cells as well as leukemic stem cells. Co-blockade of Tim-3 and PD-1 can result in reduced tumor progression in preclinical models and can improve antitumor T-cell responses in cancer patients (152).

Sabatolimab (MBG453) is a high-affinity, humanized, IgG4 (S228P) antibody targeting TIM-3, being tested together with HMAs in MDS and AML patients. The first report of the phase IB NCT03066648 was presented at ASH 2019 (149). AML patients were treated with either decitabine 20 mg/m2 IV days 1–5 or azacytidine 75 mg/m2IV/SC days 1–7, followed by escalating dose cohorts of IV sabatolimab [240 or 400 mg Q2W (D8, D22) or 800 mg Q4W (D8)] per 28-day cycle. Twenty-nine R/R AML patients (median age 68 years) were treated with decitabine+sabatolimab, resulting in 23% of CRi (156). At ASH 2020, an update of the trial NCT03066648 was presented, with more data available for 48 newly diagnosed AML and 39 high-risk MDS patients (153).

The most common gr ≥3 TEAEs in pts with ND AML, were thrombocytopenia (45.8%), neutropenia (50%), febrile neutropenia (29.2%), anemia (27.1%), and pneumonia (10.4%). Grade 3, but no grade 4, treatment-related possible immune-mediated AEs were reported in five patients. Among 34 evaluable pts with ND AML, ORR was 41.2%: eight CR, three CRi, three PR. Median time to response was 2.1 months and estimated 6-mo DOR rate was 85.1%. (95% CI: 68–100%) (153). These results provide evidence of promising antileukemic activity for the combination of sabatolimab + HMA, and support TIM-3 as a potential therapeutic target in patients with AML or higher-risk MDS.

Magrolimab is a first-in-class investigational monoclonal antibody against CD47 and a macrophage checkpoint inhibitor that is designed to interfere with recognition of CD47 by the SIRPα receptor on macrophages, thus blocking the “*don’t eat me*” signal used by cancer cells to avoid being ingested by macrophages (154). Magrolimab is being developed in several hematologic cancers, including MDS, AML, as well as solid tumor malignancies. The U.S. FDA recently assigned Breakthrough Designation to magrolimab, in combination with azacitidine, for the treatment of adult patients with newly-diagnosed MDS including intermediate, high-, or very high-risk IPSS-R MDS. Magrolimab also received PRIME Designation for treatment of MDS from the European Medicines Agency (EMA). In the NCT03248479 clinical trial, 64 AML patients were treated with magrolimab plus azacitidine, including 47 patients with the TP53 mutation, a treatment-refractory and poor-prognosis population with published median overall survival of 5–7 months even with HMA and venetoclax based therapies. As of November 2020, 63% (n = 27/43) of patients evaluable for efficacy achieved an objective response per ELN 2017 criteria, including 42% (n = 18/43) CR, and 12% (n = 5/43) CRi. The median DOR was 9.6 months (range: 0.03 to 18.7 months) and the median time to response was 1.95 months (range: 0.95 to 5.6 months).

Specifically for patients with the TP53 mutation, 69% (n = 20/29) evaluable patients achieved a response, including 45% (n = 13/29) CR and 14% (n = 4/29) CRi. The median DOR was 7.6 months (range: 0.03 to 15.1 months). Preliminary median OS was encouraging in both TP53-wild-type patients (n = 16) at 18.9 months (95% CI: 4.34, NE) and the TP53-mutant patients (n = 47) at 12.9 months (95% CI: 8.21, 17.28). The median follow-up for TP53-wild-type and TP53-mutant patients was 12.5 and 4.7 months, respectively. Additional patients and longer follow-up in a comparative trial are needed to further characterize the survival benefit.

Treatment-related adverse events observed with over 15% incidence included anemia, fatigue, blood bilirubin increased, infusion related reaction, neutropenia, thrombocytopenia and ALT increase. Most patients were cytopenic at baseline, and importantly no significant increased cytopenias, infections or immune-related adverse events (AEs) were observed in the study. Thirty-day all-cause mortality was 4.7% (n = 3/64). Treatment discontinuation due to drug-related AE occurred in 4.7% of all patients (155). Overall the safety profile of this combination has been favorable. **Table 3** summarizes the most relevant results with immune-checkpoint and machrophage inhibitors.

**Table 3:** Checkpoint blockade.

Category	Drug name	Drug details	Antigen/target	Combination therapy	Indication (AML only)	Effects *in vitro o vivo*	Refs
**ICPI**	Ipilimumab	CTLA-4 inhibitor	CTLA-4	After allogeneic SCT; decitabine	R/R AML after prior allo-SCT	Durable CR	(142–151)
	Nivolumab	PD-1 inhibitor	PD-1	Alone; azacitidine; idarubicin and cytarabine	Maintenance in high risk secondary/therapy related AML; consolidation in relapsed AML; front line therapy in *de novo* high risk AML	Good efficacy and favorable safety profile	(144–149)
	Pembrolizumab	PD-1 inhibitor	PD-1	Alone after HDAC followed to allo-SCT; azacitidine	R/R AML; *de novo* and R/R AML, elderly patient	Good efficacy and favorable safety profile	(147, 148)
	Sabatolimab	IgG4 antibody	TIM-3	HMAs	*De novo* and R/R AML	Good efficacy and favorable safety profile	(152, 153)
	Magrolimab	Macrophage checkpoint inhibitor	SIRPα	HMA; venetoclax	Refractory AML TP53 mutated or wild-type	Good efficacy and favorable safety profile	(154, 155)

## Leukemia Vaccines

The increasing evidence of a significant impact of several immunotherapy strategies harnessing patients’ immunity against acute leukemia cells is the proof that, although a wide variety of tolerogenic mechanisms may have negatively edited anti-leukemia immune response, the latter is still active and may be elicited by the use of anti-leukemia vaccines. The results of vaccine-based studies indicate the feasibility of vaccinating AML and in most cases immunological responses were observed, supporting the proof of principle that antigen-specific immune response may be elicited in AML patients through vaccination. However, with some important clinical exceptions, the clinical results are globally unsatisfactory, with few significant clinical responses. In this scenario, the availability of novel strategies involving adoptively transferred T cells may provide the rationale for developing a new generation of anti-leukemia vaccines that may be specifically used to enhance the function and persistence of transferred T cells. Moreover, immune checkpoint and macrophage checkpoint blockade may also represent an intriguing and innovative area regarding where to position anti-leukemia vaccines under a new perspective. Although a comprehensive review of leukemia vaccines is out of the scope of this Review, herein we report the most significant contributions to the field according to the target antigens that have been used. Among them, Wilms’ tumor 1 (WT1) has probably accumulated the most relevant evidence in the clinical setting, but also other target antigens, such as Proteinase-3 (PR3), preferentially expressed antigen of melanoma (PRAME) and receptor for hyaluronic acid-mediated motility (RHAMM) have been explored and are under clinical investigation for the treatment of AML.

Wilms’ tumor 1 (WT1) is highly overexpressed in more than 90% of AML cases, being an attractive antigen to be used as target for vaccination purpose in AML (157). Identification of immunogenic WT1 epitopes, capable of eliciting an anti-leukemia cytotoxic CD8^+^ T cells was reported in 2000 (158), thus providing the rationale and preclinical background for the development of clinical studies using WT1 vaccines. In particular, in two early studies (159, 160) a WT1 peptide vaccine was capable of inducing WT1-specific cytotoxic CD8^+^ T cells, which was associated with some clinical responses, including stabilization of leukemia and achievement of MRD negativity. Importantly, vaccine therapy showed no severe side effects and was well tolerated even for elderly patients. Starting from the observation that in some AML patients from early Phase I studies, the long-term WT1 peptide vaccination resulted in prolonged MRD negativity, a new generation of studies addressed the capacity of WT1-based vaccines to prevent relapse in patients who had achieved CR after chemotherapy (161–164). The post-chemotherapy or post allo-SCT CR setting, including MRD positivity, may represent the ideal setting to exploit the capacity of cancer vaccines for the prevention of relapse and as an immunological maintenance strategy. In support of that, by using different vaccine formulations, i.e. peptide and dendritic cells, the clinical results from these studies revealed better outcomes as compared to the historical control data in patients who were capable of activating a documented WT1-specific immune response. Interestingly, a better response was also observed in patients who had an increased frequency of WT1-specific CTLs before vaccination. Along the same line, WT1 vaccine has been recently evaluated as maintenance therapy in patients who had undergone allo-SCT (165, 166). Early clinical data suggest the feasibility of such strategy, even in the setting of graft-versus-leukemia effect, along with its capacity to prevent relapse. The clinical efficacy of WT1 targeting after allo-SCT may be corroborated by the recent demonstration that a T cell receptor gene therapy targeting WT1 prevented AML relapse post-transplant (167, 168).

Proteinase 3 (P3) is a serine proteinase present in the primary granules of neutrophils. Preliminary studies demonstrated that PR3-specific CD8 T cell-responses can be elicited in patients with AML, offering the rational to exploit this antigen in leukemia vaccination strategies (169). Recently, PR1, an HLA-A2-restricted peptide derived from both proteinase 3 and neutrophil elastase, has been tested in a phase I/II peptide vaccination trial on 66 patients with myeloid neoplasms, including AML. PR1-specific immune response was seen in nine of 25 clinical responders versus three of 28 clinical non-responders (P = 0.03). suggesting that PR1 peptide vaccine may induce specific immunity correlating with clinical response (170). Based on the initial demonstration that polyclonal memory CD8+ T-cell responses to PRAME-specific peptides can be detectable in patients with different hematological malignancies, including AML (171), some other studies have addressed the capacity to elicit anti-leukemia response. Interestingly, by using an *in vitro* model with peptide-loaded allogeneic dendritic cells, PRAME-reactive and functional CTLs could be isolated from healthy individuals and maintained in culture, but not from AML patients, even when samples were collected in patients that had recently achieved a CR (172). Although not conclusive and possibly dependent on the type of strategy used to elicit anti-leukemia response, these results suggest that important differences may exist among AML patients and further studies are warranted to establish the potential of PRAME as effective target antigen for AML vaccination. A phase 1 clinical trial included 10 patients with hematological malignancies, who were vaccinated with a highly immunogenic CD8(+) T-cell epitope peptide derived from RHAMM. In seven of 10 patients, an increase of fully functional RHAMM-specific CD8(+) effector T cells was observed and some clinical responses were reported (1/3 AML patients) (173).

## Conclusions

Immunotherapy is an emerging, promising strategy in AML that will be further investigated in ongoing trials. Currently, it remains to decide its ideal setting and the biomarkers predictive for response. Several agents, used in monotherapy or combined with chemotherapy, venetoclax or HMA could provide long-term disease control and improved quality of life of AML patients. Expansion cohorts phase 3 trials of agents reviewed are planned in a near future. Combining clinical results with immunological findings, possibly coupled with the discovery of biomarkers predictive for response, will allow us to determine the best approaches to immunotherapy in AML, also in those high-risk scenarios with TP53 mutations.

## Author Contributions

Study design and coordination: AI, CC, ND, and AC. Update on bispecific antibodies: AR, FL, and GV. Update on CD33 monoclonal antibodies: MK, GM, and AI. Update on other monoclonal antibodies: EJ, FL, GV, and ND. Update on NK cells: AC. Update on CAR-T cells: AC and HK. Update on CPI: ND, AR, CC, and HK. Update on combination therapy: CD and GG-M. All authors contributed to the article and approved the submitted version.

## Conflict of Interest

The authors declare that the research was conducted in the absence of any commercial or financial relationships that could be construed as a potential conflict of interest.
